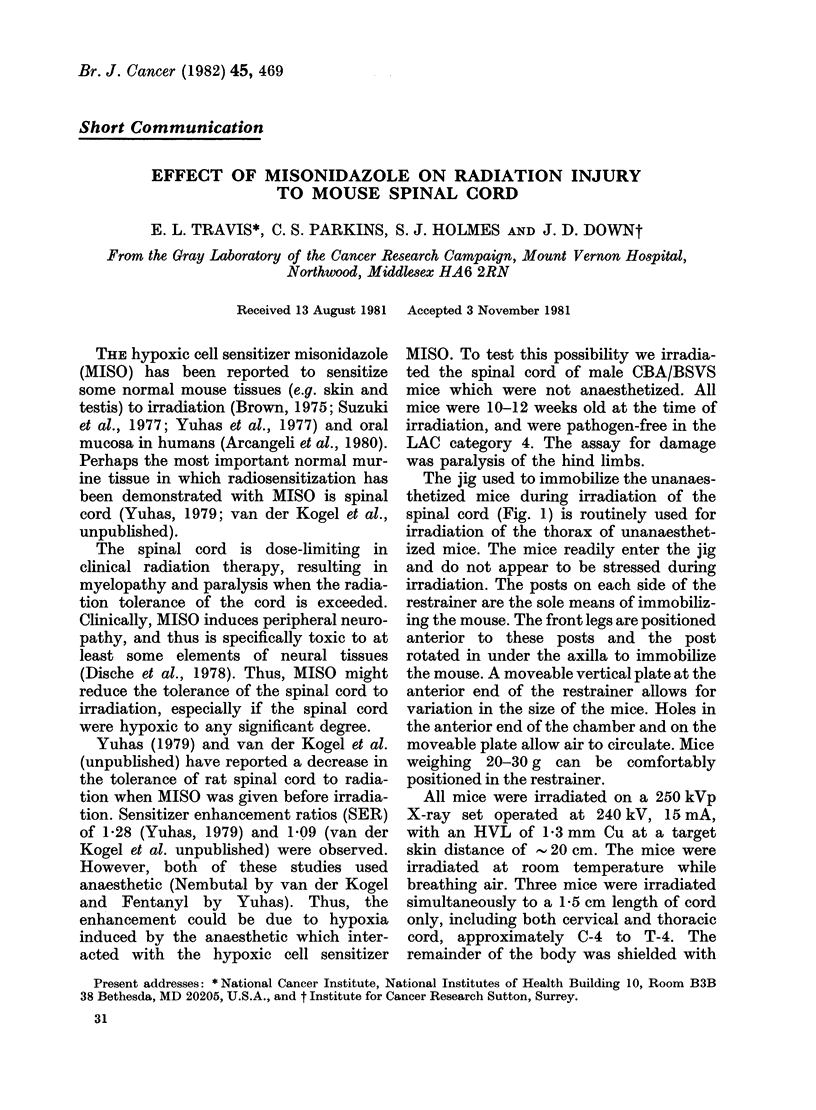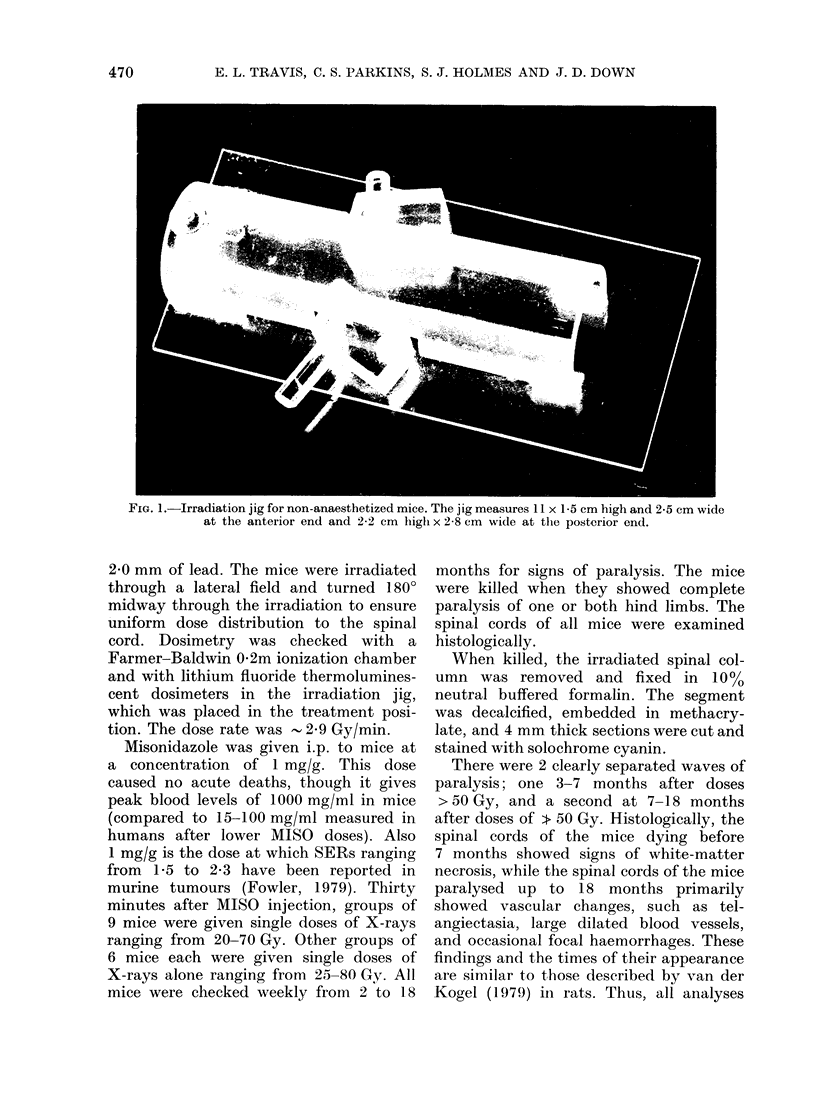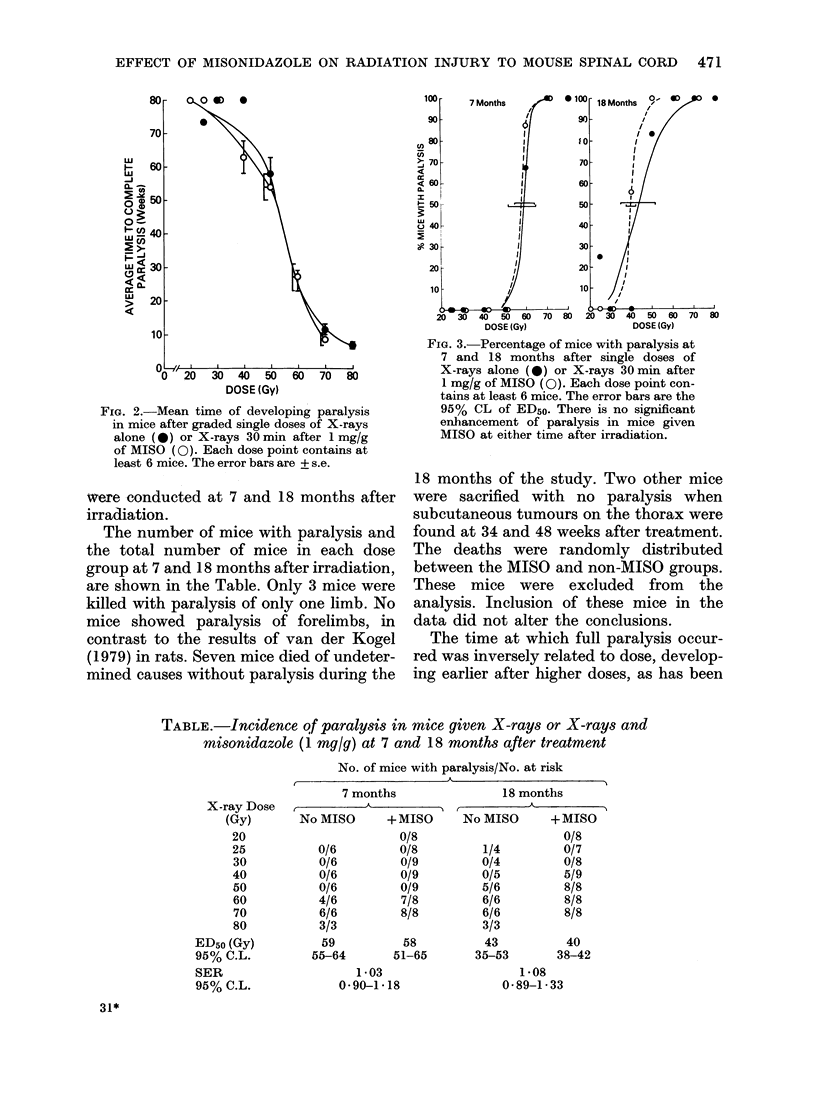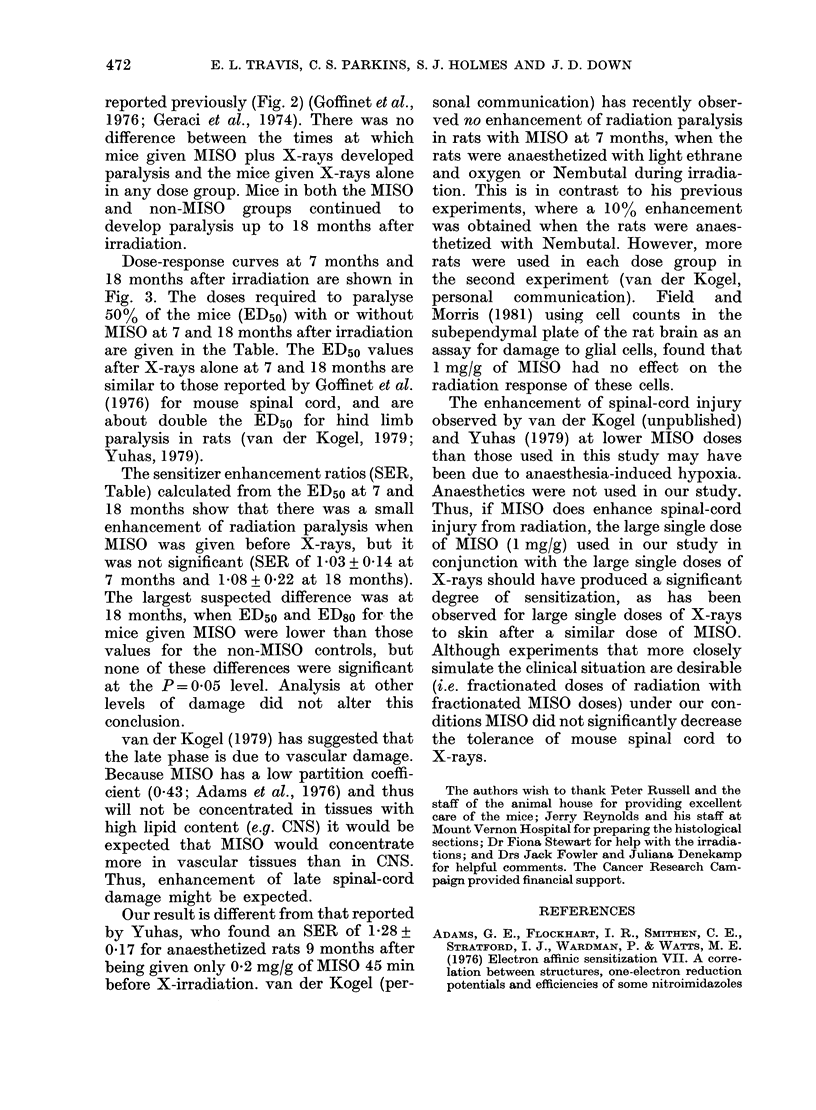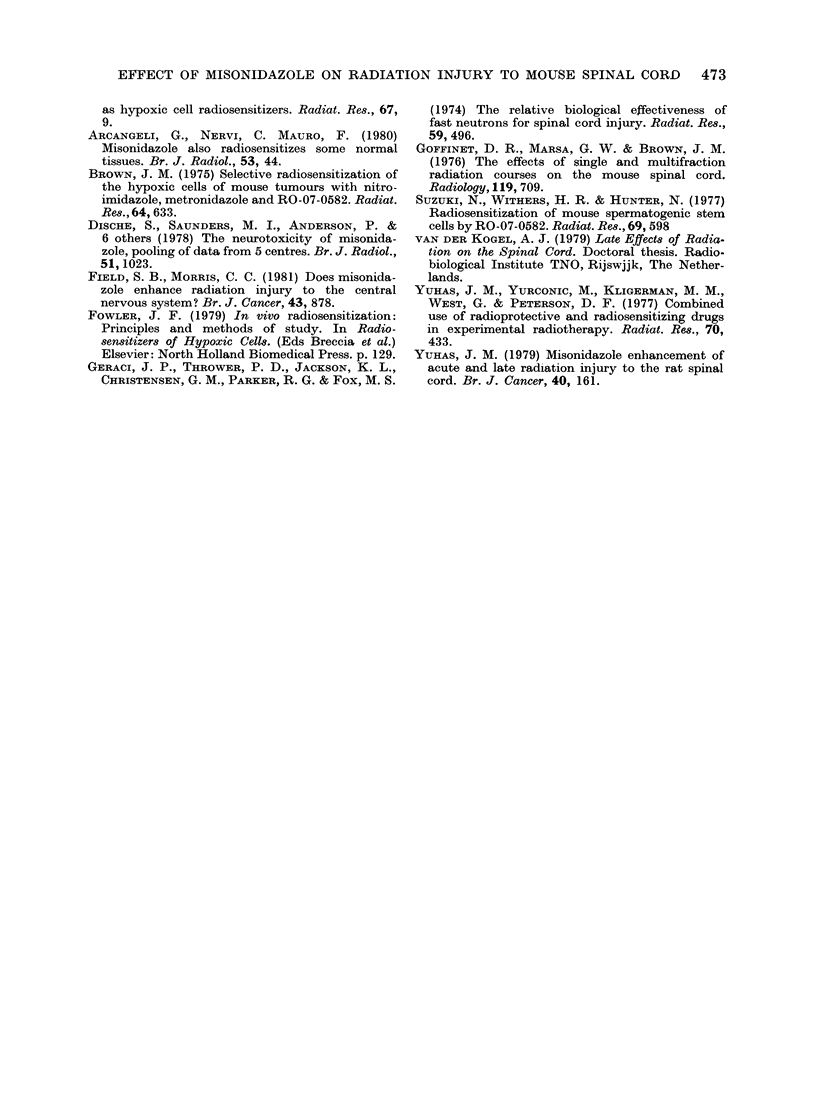# Effect of misonidazole on radiation injury to mouse spinal cord.

**DOI:** 10.1038/bjc.1982.76

**Published:** 1982-03

**Authors:** E. L. Travis, C. S. Parkins, S. J. Holmes, J. D. Down

## Abstract

**Images:**


*Br. J. Cancer* (1982) **45**, 469

***Short Communication***

### EFFECT OF MISONIDAZOLE ON RADIATION INJURY TO MOUSE SPINAL CORD

E. L. TRAVIS*, C. S. PARKINS, S. J. HOLMES AND J. D. DOWN†

*From the Gray Laboratory of the Cancer Research Campaign, Mount Vernon Hospital, Northwood, Middlesex HA6 2RN*

Received 13 August 1981 Accepted 3 November 1981

THE hypoxic cell sensitizer misonidazole (MISO) has been reported to sensitize some normal mouse tissues (*e.g.* skin and testis) to irradiation (Brown, 1975; Suzuki *et al.*, 1977; Yuhas *et al.*, 1977) and oral mucosa in humans (Arcangeli *et al.*, 1980). Perhaps the most important normal murine tissue in which radiosensitization has been demonstrated with MISO is spinal cord (Yuhas, 1979; van der Kogel *et al.*, unpublished).

The spinal cord is dose-limiting in clinical radiation therapy, resulting in myelopathy and paralysis when the radiation tolerance of the cord is exceeded. Clinically, MISO induces peripheral neuropathy, and thus is specifically toxic to at least some elements of neural tissues (Dische *et al.*, 1978). Thus, MISO might reduce the tolerance of the spinal cord to irradiation, especially if the spinal cord were hypoxic to any significant degree.

Yuhas (1979) and van der Kogel *et al.* (unpublished) have reported a decrease in the tolerance of rat spinal cord to radiation when MISO was given before irradiation. Sensitizer enhancement ratios (SER) of 1·28 (Yuhas, 1979) and 1·09 (van der Kogel *et al.* unpublished) were observed. However, both of these studies used anaesthetic (Nembutal by van der Kogel and Fentanyl by Yuhas). Thus, the enhancement could be due to hypoxia induced by the anaesthetic which interacted with the hypoxic cell sensitizer MISO. To test this possibility we irradiated the spinal cord of male CBA/BSVS mice which were not anaesthetized. All mice were 10–12 weeks old at the time of irradiation, and were pathogen-free in the LAC category 4. The assay for damage was paralysis of the hind limbs.

The jig used to immobilize the unanaesthetized mice during irradiation of the spinal cord (Fig. 1) is routinely used for irradiation of the thorax of unanaesthetized mice. The mice readily enter the jig and do not appear to be stressed during irradiation. The posts on each side of the restrainer are the sole means of immobilizing the mouse. The front legs are positioned anterior to these posts and the post rotated in under the axilla to immobilize the mouse. A moveable vertical plate at the anterior end of the restrainer allows for variation in the size of the mice. Holes in the anterior end of the chamber and on the moveable plate allow air to circulate. Mice weighing 20–30 g can be comfortably positioned in the restrainer.

All mice were irradiated on a 250 kVp X-ray set operated at 240 kV, 15 mA, with an HVL of 1·3 mm Cu at a target skin distance of ~20 cm. The mice were irradiated at room temperature while breathing air. Three mice were irradiated simultaneously to a 1·5 cm length of cord only, including both cervical and thoracic cord, approximately C-4 to T-4. The remainder of the body was shielded with

Present addresses: * National Cancer Institute, National Institutes of Health Building 10, Room B3B 38 Bethesda, MD 20205, U.S.A., and † Institute for Cancer Research Sutton, Surrey.

FIG. 1.—Irradiation jig for non-anaesthetized mice. The jig measures 11 × 1·5 cm high and 2·5 cm wide at the anterior end and 2·2 cm high × 2·8 cm wide at the posterior end.

2·0 mm of lead. The mice were irradiated through a lateral field and turned 180° midway through the irradiation to ensure uniform dose distribution to the spinal cord. Dosimetry was checked with a Farmer–Baldwin 0·2m ionization chamber and with lithium fluoride thermoluminescent dosimeters in the irradiation jig, which was placed in the treatment position. The dose rate was ~2·9 Gy/min.

Misonidazole was given i.p. to mice at a concentration of 1 mg/g. This dose caused no acute deaths, though it gives peak blood levels of 1000 mg/ml in mice (compared to 15–100 mg/ml measured in humans after lower MISO doses). Also 1 mg/g is the dose at which SERs ranging from 1·5 to 2·3 have been reported in murine tumours (Fowler, 1979). Thirty minutes after MISO injection, groups of 9 mice were given single doses of X-rays ranging from 20–70 Gy. Other groups of 6 mice each were given single doses of X-rays alone ranging from 25–80 Gy. All mice were checked weekly from 2 to 18 months for signs of paralysis. The mice were killed when they showed complete paralysis of one or both hind limbs. The spinal cords of all mice were examined histologically.

When killed, the irradiated spinal column was removed and fixed in 10% neutral buffered formalin. The segment was decalcified, embedded in methacrylate, and 4 mm thick sections were cut and stained with solochrome cyanin.

There were 2 clearly separated waves of paralysis; one 3–7 months after doses >50 Gy, and a second at 7–18 months after doses of $\ngtr$ 50 Gy. Histologically, the spinal cords of the mice dying before 7 months showed signs of white-matter necrosis, while the spinal cords of the mice paralysed up to 18 months primarily showed vascular changes, such as telangiectasia, large dilated blood vessels, and occasional focal haemorrhages. These findings and the times of their appearance are similar to those described by van der Kogel (1979) in rats. Thus, all analyses

Fig. 2.—Mean time of developing paralysis in mice after graded single doses of X-rays alone (●) or X-rays 30 min after 1 mg/g of MISO (○). Each dose point contains at least 6 mice. The error bars are ± s.e.

Fig. 3.—Percentage of mice with paralysis at 7 and 18 months after single doses of X-rays alone (●) or X-rays 30 min after 1 mg/g of MISO (○). Each dose point contains at least 6 mice. The error bars are the 95% CL of $ED_{50}$. There is no significant enhancement of paralysis in mice given MISO at either time after irradiation.

were conducted at 7 and 18 months after irradiation.

The number of mice with paralysis and the total number of mice in each dose group at 7 and 18 months after irradiation, are shown in the Table. Only 3 mice were killed with paralysis of only one limb. No mice showed paralysis of forelimbs, in contrast to the results of van der Kogel (1979) in rats. Seven mice died of undetermined causes without paralysis during the 18 months of the study. Two other mice were sacrified with no paralysis when subcutaneous tumours on the thorax were found at 34 and 48 weeks after treatment. The deaths were randomly distributed between the MISO and non-MISO groups. These mice were excluded from the analysis. Inclusion of these mice in the data did not alter the conclusions.

The time at which full paralysis occurred was inversely related to dose, developing earlier after higher doses, as has been

Table.—*Incidence of paralysis in mice given X-rays or X-rays and misonidazole* (1 *mg/g*) *at* 7 *and* 18 *months after treatment*

| X-ray Dose (Gy) | No. of mice with paralysis/No. at risk | | | |
|---|---|---|---|---|
| | 7 months | | 18 months | |
| | No MISO | +MISO | No MISO | +MISO |
| 20 | | 0/8 | | 0/8 |
| 25 | 0/6 | 0/8 | 1/4 | 0/7 |
| 30 | 0/6 | 0/9 | 0/4 | 0/8 |
| 40 | 0/6 | 0/9 | 0/5 | 5/9 |
| 50 | 0/6 | 0/9 | 5/6 | 8/8 |
| 60 | 4/6 | 7/8 | 6/6 | 8/8 |
| 70 | 6/6 | 8/8 | 6/6 | 8/8 |
| 80 | 3/3 | | 3/3 | |
| $ED_{50}$ (Gy) | 59 | 58 | 43 | 40 |
| 95% C.L. | 55–64 | 51–65 | 35–53 | 38–42 |
| SER | 1·03 | | 1·08 | |
| 95% C.L. | 0·90–1·18 | | 0·89–1·33 | |

reported previously (Fig. 2) (Goffinet *et al.*, 1976; Geraci *et al.*, 1974). There was no difference between the times at which mice given MISO plus X-rays developed paralysis and the mice given X-rays alone in any dose group. Mice in both the MISO and non-MISO groups continued to develop paralysis up to 18 months after irradiation.

Dose-response curves at 7 months and 18 months after irradiation are shown in Fig. 3. The doses required to paralyse 50% of the mice ($ED_{50}$) with or without MISO at 7 and 18 months after irradiation are given in the Table. The $ED_{50}$ values after X-rays alone at 7 and 18 months are similar to those reported by Goffinet *et al.* (1976) for mouse spinal cord, and are about double the $ED_{50}$ for hind limb paralysis in rats (van der Kogel, 1979; Yuhas, 1979).

The sensitizer enhancement ratios (SER, Table) calculated from the $ED_{50}$ at 7 and 18 months show that there was a small enhancement of radiation paralysis when MISO was given before X-rays, but it was not significant (SER of $1{\cdot}03 \pm 0{\cdot}14$ at 7 months and $1{\cdot}08 \pm 0{\cdot}22$ at 18 months). The largest suspected difference was at 18 months, when $ED_{50}$ and $ED_{80}$ for the mice given MISO were lower than those values for the non-MISO controls, but none of these differences were significant at the $P = 0{\cdot}05$ level. Analysis at other levels of damage did not alter this conclusion.

van der Kogel (1979) has suggested that the late phase is due to vascular damage. Because MISO has a low partition coefficient (0·43; Adams *et al.*, 1976) and thus will not be concentrated in tissues with high lipid content (*e.g.* CNS) it would be expected that MISO would concentrate more in vascular tissues than in CNS. Thus, enhancement of late spinal-cord damage might be expected.

Our result is different from that reported by Yuhas, who found an SER of $1{\cdot}28 \pm 0{\cdot}17$ for anaesthetized rats 9 months after being given only 0·2 mg/g of MISO 45 min before X-irradiation. van der Kogel (personal communication) has recently observed *no* enhancement of radiation paralysis in rats with MISO at 7 months, when the rats were anaesthetized with light ethrane and oxygen or Nembutal during irradiation. This is in contrast to his previous experiments, where a 10% enhancement was obtained when the rats were anaesthetized with Nembutal. However, more rats were used in each dose group in the second experiment (van der Kogel, personal communication). Field and Morris (1981) using cell counts in the subependymal plate of the rat brain as an assay for damage to glial cells, found that 1 mg/g of MISO had no effect on the radiation response of these cells.

The enhancement of spinal-cord injury observed by van der Kogel (unpublished) and Yuhas (1979) at lower MISO doses than those used in this study may have been due to anaesthesia-induced hypoxia. Anaesthetics were not used in our study. Thus, if MISO does enhance spinal-cord injury from radiation, the large single dose of MISO (1 mg/g) used in our study in conjunction with the large single doses of X-rays should have produced a significant degree of sensitization, as has been observed for large single doses of X-rays to skin after a similar dose of MISO. Although experiments that more closely simulate the clinical situation are desirable (*i.e.* fractionated doses of radiation with fractionated MISO doses) under our conditions MISO did not significantly decrease the tolerance of mouse spinal cord to X-rays.

The authors wish to thank Peter Russell and the staff of the animal house for providing excellent care of the mice; Jerry Reynolds and his staff at Mount Vernon Hospital for preparing the histological sections; Dr Fiona Stewart for help with the irradiations; and Drs Jack Fowler and Juliana Denekamp for helpful comments. The Cancer Research Campaign provided financial support.

### REFERENCES

Adams, G. E., Flockhart, I. R., Smithen, C. E., Stratford, I. J., Wardman, P. & Watts, M. E. (1976) Electron affinic sensitization VII. A correlation between structures, one-electron reduction potentials and efficiencies of some nitroimidazoles as hypoxic cell radiosensitizers. *Radiat. Res.*, **67**, 9.

ARCANGELI, G., NERVI, C. MAURO, F. (1980) Misonidazole also radiosensitizes some normal tissues. *Br. J. Radiol.*, **53**, 44.

BROWN, J. M. (1975) Selective radiosensitization of the hypoxic cells of mouse tumours with nitroimidazole, metronidazole and RO-07-0582. *Radiat. Res.*, **64**, 633.

DISCHE, S., SAUNDERS, M. I., ANDERSON, P. & 6 others (1978) The neurotoxicity of misonidazole, pooling of data from 5 centres. *Br. J. Radiol.*, **51**, 1023.

FIELD, S. B., MORRIS, C. C. (1981) Does misonidazole enhance radiation injury to the central nervous system? *Br. J. Cancer*, **43**, 878.

FOWLER, J. F. (1979) *In vivo* radiosensitization: Principles and methods of study. In *Radiosensitizers of Hypoxic Cells*. (Eds Breccia *et al.*) Elsevier: North Holland Biomedical Press. p. 129.

GERACI, J. P., THROWER, P. D., JACKSON, K. L., CHRISTENSEN, G. M., PARKER, R. G. & FOX, M. S. (1974) The relative biological effectiveness of fast neutrons for spinal cord injury. *Radiat. Res.*, **59**, 496.

GOFFINET, D. R., MARSA, G. W. & BROWN, J. M. (1976) The effects of single and multifraction radiation courses on the mouse spinal cord. *Radiology*, **119**, 709.

SUZUKI, N., WITHERS, H. R. & HUNTER, N. (1977) Radiosensitization of mouse spermatogenic stem cells by RO-07-0582. *Radiat. Res.*, **69**, 598

VAN DER KOGEL, A. J. (1979) *Late Effects of Radiation on the Spinal Cord.* Doctoral thesis. Radiobiological Institute TNO, Rijswjjk, The Netherlands.

YUHAS, J. M., YURCONIC, M., KLIGERMAN, M. M., WEST, G. & PETERSON, D. F. (1977) Combined use of radioprotective and radiosensitizing drugs in experimental radiotherapy. *Radiat. Res.*, **70**, 433.

YUHAS, J. M. (1979) Misonidazole enhancement of acute and late radiation injury to the rat spinal cord. *Br. J. Cancer*, **40**, 161.